# Association of *DCDC2* Polymorphisms with Normal Variations in Reading Abilities in a Chinese Population

**DOI:** 10.1371/journal.pone.0153603

**Published:** 2016-04-21

**Authors:** Yuping Zhang, Jun Li, Shuang Song, Twila Tardif, Margit Burmeister, Sandra M. Villafuerte, Mengmeng Su, Catherine McBride, Hua Shu

**Affiliations:** 1 Department of Psychology, Chengdu Medical College, Chengdu, China; 2 ResearchCenter for Applied Psychology of Sichuan, Chengdu Medical College, Chengdu, China; 3 State Key Laboratory of Cognitive Neuroscience and Learning, Beijing Normal University, Beijing, China; 4 Department of Psychology, University of Michigan, Ann Arbor, Michigan, United States of America; 5 Molecular and Behavioral Neuroscience Institute, University of Michigan, Ann Arbor, Michigan, United States of America; 6 Department of Psychiatry, University of Michigan, Ann Arbor, Michigan, United States of America; 7 Department of Human Genetics, University of Michigan, Ann Arbor, Michigan, United States of America; 8 Department of Psychology, The Chinese University of Hong Kong, Hong Kong, China; 9 State Key Laboratory of Cognitive Neuroscience and Learning & IDG/McGovern Institute for Brain Research, Beijing Normal University, Beijing, China; University of Leicester, UNITED KINGDOM

## Abstract

The doublecortin domain-containing 2 (*DCDC2*) gene, which is located on chromosome 6p22.1, has been widely suggested to be a candidate gene for dyslexia, but its role in typical reading development over time remains to be clarified. In the present study, we explored the role of *DCDC2* in contributing to the individual differences in reading development from ages 6 to 11 years by analysing data from 284 unrelated children who were participating in the Chinese Longitudinal Study of Reading Development (CLSRD). The associations of eight single nucleotide polymorphisms (SNPs) in *DCDC2* with the latent intercept and slope of children’s reading scores were examined in the first step. There was significant support for an association of rs807724 with the intercept for the reading comprehension measure of reading fluency, and the minor “G” allele was associated with poor reading performance. Next, we further tested the rs807724 SNP in association with the reading ability at each tested time and revealed that, in addition to significant associations with the two main reading measures (reading fluency and Chinese character reading) over multiple testing occasions, this SNP also showed associations with reading-related cognitive skills, including morphological production, orthographic judgment and phonological processing skills (rapid number naming, phoneme deletion, and tone detection). This study provides support for *DCDC2* as a risk gene for reading disability and suggests that this gene is also operative for typical reading development in the Han population.

## Introduction

Developmental dyslexia, or reading disability, is one of the most common neurobehavioral disorders and is characterized by impairments in learning to read, despite normal intelligence and adequate educational opportunities [1]. The prevalence of developmental dyslexia was estimated to be 5–10% in school-aged children across different populations, including Chinese [2–4]. Although the pathophysiology of the disorder is still not clear, there is strong evidence for genetic contributions to the risk for dyslexia (or reading abilities), with heritability ranging from 0.4 to 0.7 across studies [5–7]. Recently, doublecortin domain-containing protein 2 (*DCDC2*) [8–10], which is located on chromosome 6p22.1 [11], has been widely accepted to be one of the candidate genes for dyslexia. Functional studies have revealed the down-regulation of *DCDC2*interrupts neuronal migration in the developing cortex in rats [10, 12] and this gene also has been found to express in brain regions related to fluent reading, such as inferior and medial temporal cortex[10].

The association between *DCDC2* and dyslexia has been supported by several independent studies [10, 13]. Deffenbacher et al. [8] firstly reported this in analyses of severe dyslexics selected from a sample of 349 Colorado twin families. Of the 13 single nucleotide polymorphisms (SNPs) showing significant associations with reading-related phenotypes, eight were located within *DCDC2*. Another study [10] tested 147 SNPs in a sample of 153 dyslexic families from Colorado and reported a nominal significance for 37 SNPs, 11 of which were located in *DCDC2*. In the same study, two of the five SNPs that yielded a *p* value ≤0.01 were located in *DCDC2*. Several follow-up studies using the case-control design [14, 15] have also reported positive associations for SNPs within *DCDC2* with dyslexia. In addition to dyslexia, *DCDC2* has also been reported to contribute to reading abilities in the general population [9, 16, 17]. Scerri et al. [17] suggested that *DCDC2* was associated with reading-related traits in a large general sample (n = 3,725) from southwest England. It also yielded significant results in further analyses for dyslexia. Together, the above findings supported the notion that *DCDC2* plays an important role in reading ability and/or disabilities.

Moreover, of all of the SNPs that showed associations with dyslexia or reading abilities, rs807724 was among the most replicated. Out of the nine published studies, five reported positive associations of rs807724 with the cognitive skills underling reading ability [8, 10, 14, 15, 17]. Deffenbacher et al. [8] reported an association of rs807724 with the measure of orthographic coding, as well as associations of three haplotypes involving rs807724 with phonological awareness and/or phonological decoding. In a large longitudinal sample (n = 3,725), Scerri et al. [17] reported significant associations between rs807724 and both reading and spelling. In another study, Meng et al. [10] reported that rs807724 was strongly associated with general reading ability. In addition to rs807724, two other *DCDC2* SNPs, rs1419228 and rs793862, have also been widely investigated. In analyses of samples from the U.S. and Australia, significant associations were found between rs1419228 and several different reading-related cognitive skills, including orthographic coding; phonological decoding; reading for irregular, regular and non-words; and spelling for irregular and regular words [8, 9]. For the SNP rs793862, previous studies not only supported its association with dyslexia [15, 17, 18], but also with reading-related cognitive skills, including orthographic coding, phonological decoding, reading and spelling [8, 17]. In addition to the three above-mentioned SNPs, studies have also reported positive associations for six other SNPs within *DCDC2*, namely, rs793862, rs1770909, rs807701, rs1091047, rs2753912, and rs10498720 [11, 13, 15, 19].

We must mention that most of the previous studies on *DCDC2* were performed in Caucasian populations that use alphabetic languages [8, 9]. Noticeably, alphabetic languages are generally characterized by pronounceable letter strings. In contrast, words in the Chinese written language are characterized by a squared internal structure composed of different components corresponding with different morphemes and are less directly related to phonology [20–22]. Only one study [23] has investigated the role of *DCDC2* in reading Chinese in the Han population; however, they failed to find a positive association after Bonferroni correction. Thus, the first goal of the present study was to examine the associations between *DCDC2* and reading development in a sample of Chinese children who were followed throughout their primary school years. To do so, we examined all eight of the SNPs that have been previously reported to be associated with reading [8, 9, 15, 17]. Two tests of reading fluency (RF) and Chinese character reading (CCR) were used to measure children’s general reading abilities. In addition, we also included measures of reading-related cognitive skills, including phonological processing abilities, morphological awareness, and orthographic skills [24, 25].

Notably, recent twin studies have suggested that the rate of growth in children’s reading abilities are also heritable [26–29]. For example, Petrill et al. [29] investigated the genetic influences on children’s reading growth between the first and third grade in 283 twin pairs and reported significant genetic contributions to the initial status (i.e., intercept)of reading abilities. In another study, Christopher et al. [27] followed reading development for a group of 487 twin pairs (between post-kindergarten and fourth grade) and found that the growth (i.e., slope) in reading abilities over this period and initial reading status were both heritable. Our second goal was thus to determine whether the *DCDC2* gene polymorphisms impact longitudinal reading differences in both initial reading skills (i.e., intercept) and subsequent reading growth (i.e., slope).

## Materials and Methods

### Participants

We used data from a general population cohort of 284 (55.6% boys) unrelated Beijing-born children who were initially recruited into a norming study for the Chinese Communicative Development Inventory (CCDI) in 2000. All children were native Mandarin speakers with normal IQs and none of them reported mental, physical, or sensory difficulties on their health care records. Informed written consent was obtained from the parents of all of the children. Ethical approval for the present study was obtained from the Institutional Review Board (IRB) of Beijing Normal University Imaging Center for Brain Research and the State Key Laboratory of Cognitive Neuroscience and Learning.

### Phenotypic measurements

All 284 children were tested annually on a variety of tasks as part of an ongoing longitudinal study. The selection of tasks for the present study was based on the criterion that each had been found to be predictive of individual differences in Chinese children’s reading abilities and disabilities [4, 30, 31] and had been administered for at least three years to allow for analyses of association over time. Two main reading measures of reading fluency (RF) and single Chinese character reading(CCR), which was used to measure children’s reading abilities from the time they received formal education in primary school, were both included in the present study. Based on a previous study in Germany [32], the RF test [31] was designed to measure the children’s fluent reading comprehension between ages 7 and 11. The CCR test [4], which is comparable with tests of single word reading that are commonly used in genetic association studies of dyslexia for English and other languages [3], was used to measure the children’s reading accuracy when the children were 7, 8, 9, 10, and 11 years old. In addition, five tests were used to measure the children’s cognitive skills in three domains, including morphological awareness (morphological production, MP), orthographic skills (orthographic judgments, OJ), and phonological processing abilities (rapid number naming, RAN, phoneme deletion, PD, and tone detection, TD). Specifically, MP [4] was use to assess the children’s morphological awareness, namely, the ability to discriminate homographs and different meanings of one character, between 7 and 9 years old. The test of orthographic awareness, OJ [22], which was similar to the test of orthographic coding [8], was designed to measure the children’s ability to identify illegal characters between 6 and 8 years old. The rapid number naming task (RAN) [4] was used to measure the ability of rapid automatized naming when the children were 6, 7, 8 and 9 years old. PD [4], which was comparable with the measure of phonological awareness that is commonly used in English [8],was used to assess the ability to isolate and manipulate phonemes in speech between 7 and 9 years old. Moreover, the TD [33] test used the odd ball out format and was used to measure the ability to identify incongruent tones in speech in children between 7 and 9 years old [33]. All of the above-mentioned reading and cognitive measures have been successfully used in previous studies investigating Chinese children’s reading abilities [4, 22, 31].

The descriptive statistics for all of the quantitative phenotypes of each measurement are presented in S1 Table. There was an obvious increase in the grades for the RAN, MP, OJ, CCR, and RF measures, with no floor or ceiling effects. S2 Table presents the correlation coefficients between phenotypic measures; most are significantly correlated, except for the correlations between orthographic skills and phonological processing abilities in 6-year-old children. The two main reading measures, RF and CCR, are strongly correlated across all of the tested time points (all correlation coefficients ≥ 0.50, indicating moderate to high correlations). Moreover, all of the reading-related cognitive skills in children from 6 to 9 years old were significantly correlated with both reading measures (all correlation coefficients ≥ 0.12, *p*s ≤ 0.05). The cognitive skills within specific reading domains (i.e., the various measures of phonological processing abilities) were also significantly correlated (all correlation coefficients ≥ 0.17).

### Genotyping

The eight SNPs (rs1419228, rs793862, rs1770909, rs807701, rs807724, rs1091047, rs2753912, rs10498720) that showed significant associations in the previous studies were genotyped using the MassArray system (Sequenom, Inc., San Diego, CA, USA). Assays were designed using MassARRAY Assay Design (version 3.1) software (Sequenom Inc.) and typed using iPLEX chemistry on Complete iPLEX^®^ Gold Genotyping Reagent Set 384. Forward and reverse PCR primers and primer extension probes were purchased from Sangon Biotech Co., Ltd. (Shanghai, China). Genotyping was carried out in standard 384-wellplates with 20.0 ng genomic DNA used per sample. Allele calls were reviewed using TYPER Analyzer 3.3 (Sequenom Inc.) to evaluate assay quality.The sample success rates for all eight SNPs were at least 98%. Ten percent of all of our samples were genotyped twice, and the reproducibility of the genotyping was 100%.

### Statistical analyses

The PLINK program, version 1.07 [34], was used to carry out the Hardy-Weinberg Equilibrium (HWE) test of all eight of the SNPs and to examine the associations between the SNPs and the quantitative measures in the first step. In accord with previous studies [17], additive models were considered and were evaluated against the null hypothesis of no association. Variables were assessed for normality and association between SNPs and association with those skewed variables were tested using Kruskal Wallis Test. Additionally, in case that the associations were influenced by outliers, we reanalyzed the genotypic effect of rs807724 on reading phenotypes at each test point with outliers being exclude (S4 Table). The linkage disequilibrium of the SNPs was estimated using Haploview Version 4.2 [35]. The SPSS 20.0 program was used to perform descriptive statistics and Pearson’s correlations.

The goal of the present study was to examine genetic influences on reading abilities. Specifically, we examined SNP associations with individual variations in reading development for both the RF and CCR measures. The initial level of performance and the subsequent rate of growth in reading were estimated using latent growth modelling [36]. The models were run separately for each phenotype using the raw score in Mplus [37]. The detailed descriptions of the latent growth modelling are found in the S1 Methods. In the second step, we estimated the genetic contributions of the SNPs to both reading and reading-related cognitive skills for each specific time point.

In all of the analyses, both the age at Assessment 1 and sex were specified as covariates. We corrected for the number of independent SNPs as determined by SNPSpD [38], which yielded a significant level of 0.01 (0.05/5) after Bonferroni correction in the analyses of the development of two main reading phenotypes. As only the SNPs that showed significance in the first step were included in the following steps, we only corrected for the number of significant SNPs, namely, one, with a significance level of 0.05 in the analyses with reading and reading-related cognitive skills for each time point. As all of the quantitative phenotypes were highly correlated over time (see S2 Table), we did not correct for multiple tests in the phenotypes. Statistical power analysis was undertaken using G*power [39].On the basis of our sample size, we were able to detect an anticipated effect (F^2^) of 0.042/0.028 (or R^2^ = 0.040/0.026) with80% power (α set to 0.01/0.05).

## Results

No deviation from the HWE was found for any of the eight analysed SNPs (see Table 1). Inter-marker linkage disequilibrium (LD) was assessed in Haploview (version 4.2) [35] and suggested a low to moderate LD across *DCDC2* (Table 1), with some correlated SNPs. The demographic factors, including age, gender, and educational level, were comparable across the different genotype groups for all eight of the SNPs (all *p* values > 0.05).

**Table 1:** Linkage disequilibrium within *DCDC2*.

SNP	Minor/Major	Genotype	MAF	rs1419228	rs793862	rs1770909	rs807701	rs807724	rs1091047	rs2753912	rs10498720
rs1419228	G/A	1/25/258	0.048	--	0.748	0.683	0.677	0.692	1.000	0.524	0.884
rs793862	A/G	43/125/116	0.372	0.039	--	0.982	0.375	1.000	0.832	0.523	0.613
rs1770909	A/G	30/111/141	0.303	0.011	0.766	--	0.963	0.772	0.916	0.537	0.651
rs807701	C/T	14/110/160	0.243	0.073	0.026	0.139	--	0.976	0.954	0.817	0.467
rs807724	G/A	0/32/252	0.056	0.421	0.079	0.016	0.174	--	0.913	0.661	0.430
rs1091047	C/G	7/93/184	0.188	0.010	0.099	0.095	0.691	0.009	--	0.827	0.499
rs2753912	G/A	64/138/82	0.468	0.011	0.153	0.129	0.163	0.019	0.127	--	0.687
rs10498720	C/A	13/86/185	0.197	0.009	0.059	0.053	0.015	0.002	0.013	0.133	--

Note: MAF, minor allele frequency.

D’ values are shown in the upper half of the table, r^2^ values in the lower half of the table.

### Genetic-growth association estimates

**Fig 1:**
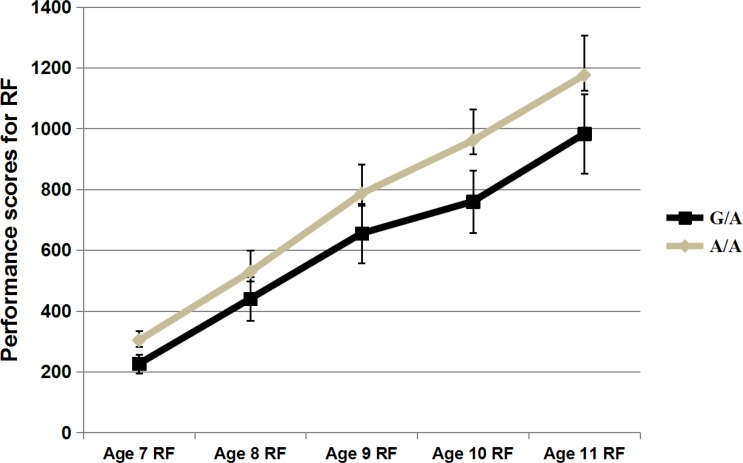
Performance of different genotypic groups of rs807724 in reading fluency (RF). *N* = 0, 32, and 252 for the three genotypes of G/G, G/A, and A/A respectively. X-lab = test time (in years), Y-lab = performance scores for reading fluency(RF).

**Fig 2:**
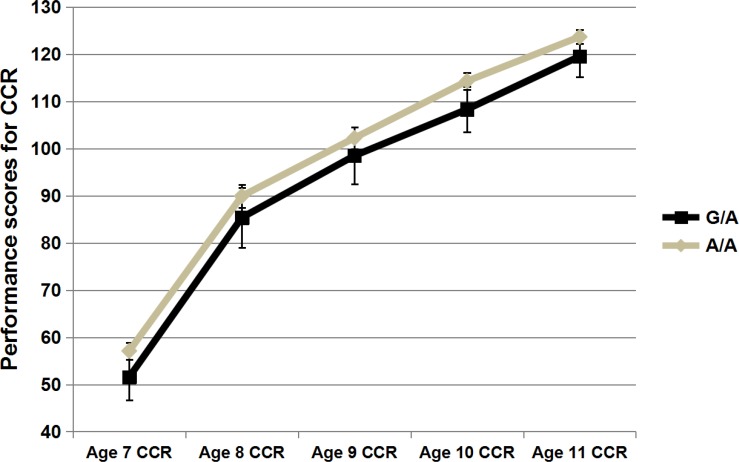
Performance of different genotypic groups of rs807724 in Chinese character reading (CCR). *N* = 0, 32, and 252 for the three genotypes of G/G, G/A, and A/A respectively. X-lab = test time (in years), Y-lab = performance scores for Chinese character reading(CCR).

The initial level of performance and the subsequent rate of growth were estimated using latent growth modelling [36] to describe children’s reading development. The estimated mean intercept, slope and model fitting estimates are presented in S3 Table. Of all eight SNPs tested for association with both the intercept and slope in the present study, association signals were only found between rs807724 and the intercept of RF (intercept, β = -0.58, *P* = 0.002) (Fig 1 & Table 2). Compared with the G/A group, the A/A group showed a higher intercept for reading fluency across the time period (between ages 7 and 11 years) tested in our study. Though only significant at the nominal level, associations between rs807724 and slope for RF, intercept for CCR showed similar trend as the A/A group showed steeper slope or better performance than the G/A group (Fig 2 & Table 2). In addition, association trends also were found between rs1419228 and both the intercept and slope for RF, with the A/A group performing better than G/- group. No association was found for the six other SNPs. Moreover, only the association between rs807724 and the intercept for RF survived Bonferroni corrections. Thus, the following analyses were only performed for rs807724.

**Table 2:** Association of *DCDC2* with reading development.

	RF								CCR							
	Latent intercept				Latent slope				Latent intercept				Latent slope			
	BETA^a^	β^b^^,^^c^	SE	*P*	BETA	β	SE	*P*	BETA	β	SE	*P*	BETA	β	SE	*P*
rs1419228	-69.620	-0.449	0.193	0.021*	-30.120	-0.450	0.189	0.018*	-30.120	-0.324	0.176	0.068	0.848	0.322	0.192	0.094
rs793862	-17.570	-0.113	0.085	0.183	-0.541	-0.008	0.083	0.923	-0.541	-0.001	0.077	0.991	-0.018	-0.007	0.084	0.935
rs1770909	-5.584	-0.036	0.089	0.686	5.575	0.083	0.087	0.340	5.575	0.043	0.081	0.594	-0.035	-0.013	0.088	0.881
rs807701	-21.170	-0.137	0.101	0.177	0.612	0.009	0.099	0.927	0.612	-0.067	0.092	0.467	0.126	0.048	0.100	0.633
rs807724	-90.170	-0.582	0.185	0.002**	-30.800	-0.460	0.182	0.012*	-30.800	-0.435	0.169	0.011*	0.719	0.273	0.186	0.142
rs1091047	7.959	0.051	0.112	0.646	11.520	0.172	0.109	0.116	11.520	0.082	0.102	0.419	-0.129	-0.049	0.111	0.657
rs2753912	18.100	0.117	0.082	0.157	-0.957	-0.014	0.081	0.860	-0.957	0.101	0.075	0.179	-0.200	-0.076	0.082	0.353
rs10498720	9.032	0.058	0.105	0.578	0.727	0.011	0.103	0.916	0.727	0.024	0.095	0.799	-0.188	-0.071	0.104	0.49

*Note*. RF-Reading fluency, CCR-Chinese character reading.

a, BETA represents unstandardized regression coefficient.

b, β represents standardized regression coefficient.

c, the minor allele was specified as the reference allele for all eight SNPs.

* *P*< 0.05

**P< 0.01

### Genetic-phenotypic association estimates

Table 3 presents the results for the association analyses for rs807724 and all of the reading measures for each tested time point. For RF, significant signals were detected at all of the tested time points for children aged 7 to 11 (age 7, β = -0.595, *P* = 0.001; age 8, β = -0.432, *P* = 0.023; age 9, β = -0.442, *P* = 0.020; age 10, β = -0.621, *P* = 0.001; and age 11, β = -0.488, *P* = 0.008). The minor allele “G” was associated with poor performance in RF. For CCR, associations with rs807724 were also found for all five tested time points (age 7, β = -0.540, *P* = 0.003; age 8, β = -0.367, *P* = 0.036; age 9, β = -0.407, *P* = 0.017; age 10, β = -0.537, *P* = 0.001; and age 11, β = -0.457, *P* = 0.003).

**Table 3:** Association of *rs807724* with reading and reading-related cognitive skills.

	RF				CCR				MP							
Age	BETA^a^	β^b^^,^^c^	SE	P	BETA	β	SE	P	BETA	β	SE	P				
6	--	--	--	--	--	--	--	--	--	--	--	--				
7	-96.470	-0.595	0.185	0.001*	-7.83	-0.540	0.179	0.003*	-2.54	-0.582	0.179	0.001*				
8	-103.300	-0.432	0.188	0.023*	-7.14	-0.367	0.175	0.036*	-3.12	-0.739	0.179	0.00005*				
9	-142.000	-0.442	0.189	0.020*	-7.11	-0.407	0.169	0.017*	-1.55	-0.418	0.184	0.024*				
10	-221.500	-0.621	0.182	0.001*	-7.79	-0.537	0.155	0.001*	--	--	--	--				
11	-199.800	-0.488	0.183	0.008*	-5.62	-0.457	0.153	0.003*	--	--	--	--				
	RAN				OJ				PD^d^				TD			
Age	BETA	β	SE	P	BETA	β	SE	P	BETA	β/χ^2^	SE	P	BETA	β	SE	P
6	3.840	0.713	0.182	0.0001*	-1.830	-0.245	0.185	0.187	--	--	--	--	--	--	--	--
7	1.240	0.351	0.188	0.063	-4.120	-0.539	0.185	0.004*	--	0.226	--	0.635	-1.690	-0.342	0.186	0.068
8	1.060	0.408	0.185	0.028*	-1.610	-0.344	0.179	0.056	--	0.768	--	0.381	-1.870	-0.375	0.186	0.044*
9	0.300	0.136	0.186	0.465	--	--	--	--	-0.230	-0.128	0.182	0.483	-2.230	-0.505	0.178	0.005*

*Note*. RF-Reading fluency, CCR-Chinese character reading, MP-Morphological production, RAN-Rapid number naming, OJ-Orthography judgment, PD-Phoneme deletion, TD-Tone detection.

a, BETA represents unstandardized regression coefficient.

b, β represents standardized regression coefficient.

c, the minor allele was specified as the reference allele.

d, association with those skewed variables were using Kruskal Wallis Test

*, *P*< 0.05.

--, Not available.

We also tested the associations between rs807724 and reading-related cognitive skills (Table 3), including MP, RAN, OJ, PD, and TD. Significant associations with MP were found for all three tested time points (age 7, β = -0.582, *P* = 0.001; age 8, β = -0.739, *P* = 0.0003; and age 9, β = -0.418, *P* = 0.024) and with RAN for two out of the four tested time points (age 6, β = 0.713, *P* = 0.0002; age 8, β = 0.408, *P* = 0.028). For TD, significant associations were also found for two of the tested time points (age 8, β = -0.375, *P* = 0.044; age 9, β = -0.505, *P* = 0.005). For OJ, associations were only found for a single tested time point (age 8, β = -0.539, *P* = 0.004), while no association was found for the phenotype of PD. Importantly, and in accord with the association test for RF and CCR, the analyses with reading-related cognitive skills also supported the hypothesis that the minor allele of rs807724 is associated with poor reading performance.

## Discussion

In the present study, we tested the association of eight SNPs spanning *DCDC2* with reading development in a sample of Chinese children who were followed from 6 to 11 years old. Only one SNP, rs807724, was found to influence the initial level of children’s reading growth, with the minor “G” allele associated with poor reading performance. Further analyses reported positive associations for all of the reading and related cognitive skills measured in this study at multiple tested time points. In summary, our findings support *DCDC2* as a candidate gene for normal variations in reading abilities in the Han population.

We initially assessed the contribution of eight *DCDC2* SNPs on reading growth using the two main reading measures, RF (fluent reading comprehension) and CCR (word reading accuracy), in children who were followed between ages 6 and 11. To the best of our knowledge, this is the first study that investigated the contribution of *DCDC2* to children’s reading trajectories. We found that rs807724 was significantly associated with the intercept (initial reading level) for RF, whereas its association with the slope was only significant at the nominal level. This is consistent with previous findings that the intercept has more of a genetic component than the slope, as suggested by twin studies [26–29]. For CCR, we found similar patterns to our analyses for RF; however, rs807724 was only nominally associated with the intercept for CCR. Previous twin studies reported significant genetic contributions to the intercept for reading fluency [27], whereas the findings for the slope are not consistent. Although Christopher et al. [27] reported reading growth was heritable through children’s post-kindergarten to post-4^th^ grade, another two independent studies both failed to find positive genetic effects for the slope of reading fluency [28, 29].

In the present study, association signal was found for RF slope, though nominally, may possibly indicated a different pattern of genetic influences on the variability in growth rates for RF and CCR. One possible explanation may be that RF differs from CCR not only in the components of reading involved but also in task-relevant components that are incidental to reading *per se*. In particular, our RF measure was a timed, fluency and accuracy-based reading comprehension measure, whereas CCR measured accuracy alone. Thus, once speed is considered, individual variations in performance on the RF task may be greater than the non-speed-based CCR reading test and this difference increases as the children get older. In addition, the larger variability in performance on the RF test also raises the possibility of higher heritability estimates for growth than an untimed measure. Moreover, the lack of association for CCR on the growth rate may also partially be due to the smaller and gradually decreased variance in the children’s word reading abilities (S1 Table), which is consistent with previous reading studies [40, 41]. This lack of variance suggests that the children in our sample grew at similar rates for CCR over this developmental window.

In our analyses for independent test occasions, we found significant associations for rs807724 at all five tested time points for both RF and CCR, with the minor “G” allele always showing a worse performance. Though these results are inconsistent with a previous study investigating rs807724 in association with dyslexia in the Chinese population [23], our findings are consistent with most previous studies on this SNP. Five out of nine studies have reported a significant association between rs807724 and reading abilities in children while reading alphabetic scripts [8, 10, 14, 15, 17]. More importantly, though specific measures may possibly differ, our reading tests were comparable with those used in previous studies. Scerri et al. [17] reported a significant association between rs807724 and three reading phenotypes, including single word reading, in a large sample from southwest England (n = 3,725). And this single word reading test was similar as CCR in measuring Chinese children’s reading ability in the present study. While both Newbury et al. [14] and Wilcke et al. [15] reported positive association with qualitative reading trait, their procedure in identifying children with dyslexia was also based on the single word reading test. In addition, Deffenbacher et al. [8] reported rs807724 positively associated with the trait of orthographic coding (OC), which is similar to our orthographic test of OJ. The other two studies [9, 42] did not find a positive association, but found a pattern that is similar to the present study. One possible explanation for our inconsistency with the study on Chinese by Sun et al. [23] might be that compared with their test for quantitative trait of dyslexia, our study aimed at testing the associations with more sensitive quantitative reading traits. Additionally, it is also possible that the association strength may vary across children’s different ages due to the sensibility for each reading measures.It is also worth noting that the present study reported a relatively large effect size (R^2^ ranged between 0.14 and 0.55) for all positive associations. This may partially be explained by our research design. In replicating the *DCDC2* and reading association in a general sample, our sample also involves about 5.3% (n = 15) dyslexic cases (at least -1.5 standard deviation below grade mean on CCR), which possibly increased the variances of the phenotype measures and brought with greater effects.

We also found that rs807724 was significantly associated with all reading-related cognitive skills. The most consistent results were found for MP, a measure of morphological awareness, with a positive association found for all three consecutive test occasions between ages 7 and 9 (*P* values ranged between 0.00005 and 0.024). In addition to the stable correlations between MP and the reading measures of RF and CCR (ranging from 0.45 to 0.60) (S2 Table), morphological awareness has previously been suggested to be a core construct that is necessary for explaining the large variability in reading Chinese [4]. Strong association was found for RAN at age 6 (*P* = 0.001), with similar patterns at ages 7 and 8 (*P* = 0.063 and 0.028, respectively). One possible reason that the association of rs807724 with RAN became less robust between 6- and 9-years-old is that the individual differences on RAN performance are gradually minimized as children grew up (S1 Table) and this decreasing variability is consistent with children’s developmental rules of RAN [22, 31]. Specifically, the larger variance for RAN at age 6 generally indicates a higher statistical power. Thus, with our limited sample size, it was easier to find a significant result at age 6 (larger variances) than the other time points (smaller variances). Previous studies also tested rs807724 in association with cognitive skills, but they did not report on either morphological awareness or RAN [8].

In addition to MP and RAN, we also found positive associations for OJ and TD, but only at certain ages. In accord with the association between rs807724 and OJ (a measure of orthographic awareness) found in the present study, Deffenbacher et al. [7] also reported nominal level associations for orthographic coding, a test of orthographic awareness that measures an individual’s ability to master orthographic rules, which is comparable to our test of OJ. However, findings from other studies did not support the association between rs807724 and orthographic skills [10, 42]. Together with the associations with RAN and TD, our results indicated that the rs807724 SNP might play a role in children’s phonological processing abilities. For PD, a measure of phonological awareness, all previous attempts failed to find an association with the rs807724 SNP [8, 10, 11]. Finally, as a tonal language, the ability to discriminate different tones is a necessary and important skill for children learning to speak and read Chinese [33]. Thus, tone awareness, as measured by TD, was also examined in this study, and a significant association was reported. Noticeably, our findings for the association with TD increased between ages 7 and 9 years, and this is consistent with the characteristics of the Chinese language. Tone has always been suggested as the most difficult part in learning to read Chinese and requires much more practice [37, 43]. However, all previous studies for *DCDC2* were performed in non-tonal alphabetic languages, such as English and German. Consequently, the association with tone awareness has not been previously studied.

Although the association signals failed to survive the Bonferroni correction, we initially detected signals for rs1419228 with both the intercept and slope of reading fluency, which indicated a potential role for this SNP in the reading growth of children. In fact, two previous studies supported the role of rs1419228 in reading [8, 9], and different reading phenotypes were found for the associations. Deffenbacher et al. [8] reported the detection of significant signals between rs1419228 and two reading phenotypes (orthographic coding and phonological decoding) in a severe dyslexia sample of CLDRC twins aged 8 to19 years. In another independent sample of 522 families of adolescent twins (aged 12 to 25 years) that were not selected for reading impairment [9], a significant association between rs1419228 and reading was also reported. However, other studies [11, 44] failed to replicate these findings for rs1419228 in either German or Canadian samples. Our findings are also inconclusive; thus, the association between rs1419228 and reading-related cognitive skills are mixed and require further clarification.

Several limitations should be taken into account in interpreting the findings from the present study. First, the relatively small sample size in this study may have decreased the reliability of our findings. Second, only a limited number of *DCDC2* markers were genotyped in this sample set of children. In addition, the associations testing for growth were based on three measurement time points for reading-related cognitive skills, which precluded the examination of non-linear growth. Despite these limitations and consistent with previous reports [9, 17], the findings of the current study support the role of the dyslexia candidate gene *DCDC2* in normal variations in reading skills in a typically developing Han Chinese sample and thus provide new evidence for the association with reading development in a non-European population.

## Supporting Information

S1 Fig Latent growth model for reading fluency (RF) and Chinese character reading (CCR). (TIF)

S1 Methods Supporting information. (DOCX)

S1 Table Descriptive statistics of phenotype measures. (DOCX)

S2 Table Pearson correlations between phenotype measures. (DOCX)

S3 Table Estimated mean intercept, slope and model fitting estimates. (DOCX)

S4 Table Association of *rs807724* with reading and reading-related cognitive skills with outliers excluded. (DOCX)
