# Supplementary figures and images for: Association of DCDC2 Polymorphisms with Normal Variations in Reading Abilities in a Chinese Population
Source: PLoS One. 2016 Apr 21;11(4):e0153603. doi: 10.1371/journal.pone.0153603 (PMC4839751; doi:10.1371/journal.pone.0153603)

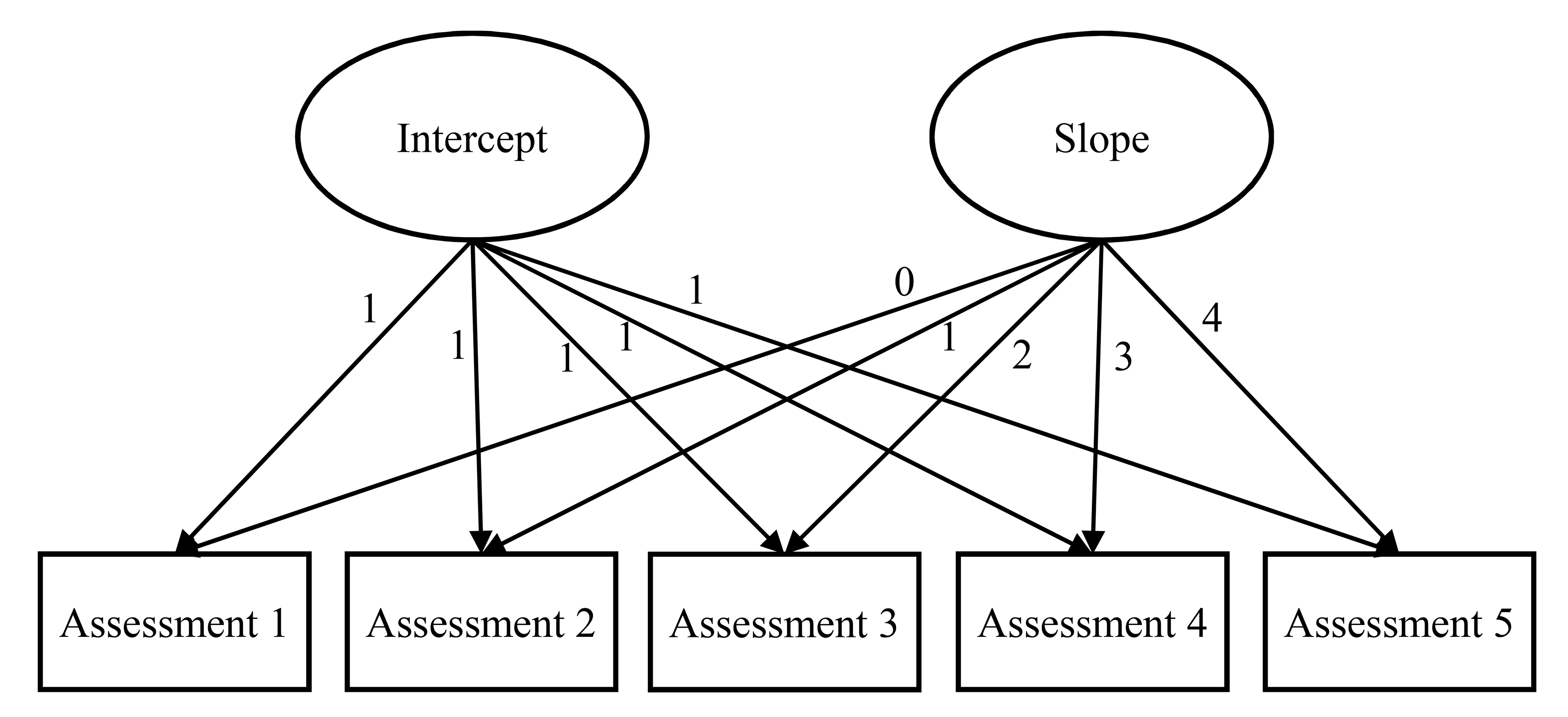

Supplement: S1 Fig — (TIF) [file pone.0153603.s001.tif]
