# Supplementary material for: Association of DCDC2 Polymorphisms with Normal Variations in Reading Abilities in a Chinese Population
Source: PLoS One. 2016 Apr 21;11(4):e0153603. doi: 10.1371/journal.pone.0153603 (PMC4839751; doi:10.1371/journal.pone.0153603)
Supplement: S1 Methods — (DOCX) [file pone.0153603.s002.docx]

**S1 Methods: Supporting Information**

**Latent Growth Estimation**

The initial performance level of the children and the subsequent rate of growth in reading were estimated for each phenotype measurement using latent growth modeling. Latent growth modeling[1] is applied with individual growth curves for each fitted participant to explain the variability around the mean intercept and mean linear growth. As presented in S1 Figure, in this single slope effect model, the mean intercept was fixed at one for each Assessment, and the time scores for the latent slopes were fixed at 0, 1, 2, 3 and 4 separately for Assessment 1, 2, 3, 4 and 5. A linear growth model with an equidistant Assessment time point was defined. As the latent slope was fixed at zero for Assessment 1, the latent intercept was defined as the initial status. Taken together, the model estimated a regression equation for each child, including an estimated initial level of latent reading performance (latent intercept) and subsequent rate of growth in latent performance across measurement occasions (latent slope).

The level 1 (individual level) model is:

$$y_{it}=\pi_{i0}+\pi_{i1}(Assesment Number-1)+e_{it}$$

$y_{it}$refers to the test score of individual $i$ at time $t$, $\pi_{i0}$ refers to the intercept at assessment 1 for individual $i$, $\pi_{i1}$ refers to the slope at assessment 1 for individual $i$, and $e_{it}$reflects error of individual $i$ at time $t$.

The level 2 model is:

$$\pi_{i0}=M_{I}+\gamma_{Ii}$$

$$\pi_{i1}=M_{S}+\gamma_{Si}$$

where$M$ refers to the weighted means of the intercept and slope parameters, respectively, and $\gamma$ to the deviation.

In the next step, the genetic association was tested using the estimated latent intercept and latent slope. It is worth mentioning that as two of the reading phenotypes were tested for more than three time points, we also tried the curvilinear growth model with estimated latent intercept, latent slope and latent quadratic growth speed for each individual. However, the quadratic speed for both single character reading and reading fluency was not significant in the test for genetic association; the R-square for each assessment only slightly increased or even decreased when compared to the linear growth model. Thus, the curvilinear growth model was abandoned.

**Statistical analyses**

The presence of outliers may possibly lead to spurious correlations. Thus, we reanalyzed the association between rs807724 and reading phenotypes with all outliers being exclude using the same model as the original analyses. Outliers [2] for phenotype measures were determined based on the following criteria separately for G/A and A/A group:

outliers = Q3 + 1.5 * (Q3 - Q1) or Q1 - 1.5 * (Q3 - Q1),

where Q1 is the 1^st^ quartile value and Q3 is the 3^rd^ quartile value.

As can be seen in S4 Table, our results didn’t change much.

**References**

1. Duncan TE, Duncan SC, Strycker LA: *An introduction to latent variable growth curve modeling: Concepts, issues, and applications*, 2nd edn. Lawrence Erlbaum Associates Inc: NJ, USA，2006.

2. Frigge, M., & Iglewicz, B. (1989). Some implementations of the boxplot. *American Statistician*, *43*(1), 50-54.
