# Supplementary material for: Association of DCDC2 Polymorphisms with Normal Variations in Reading Abilities in a Chinese Population
Source: PLoS One. 2016 Apr 21;11(4):e0153603. doi: 10.1371/journal.pone.0153603 (PMC4839751; doi:10.1371/journal.pone.0153603)
Supplement: S1 Table — (DOCX) [file pone.0153603.s003.docx]

Table S1 Descriptive statistics of phenotype measures

| Age | Phenotypic measurements | N | Mean | SD | Skewness | Kurtosis |
| --- | --- | --- | --- | --- | --- | --- |
| 7 | Reading fluency (RF) | 282 | 294.28 | 162.15 | 1.73 | 3.70 |
| 8 |  | 284 | 517.90 | 239.26 | 1.26 | 1.78 |
| 9 |  | 284 | 770.86 | 321.33 | 0.83 | 0.50 |
| 10 |  | 284 | 937.06 | 356.71 | 0.73 | 0.45 |
| 11 |  | 284 | 1153.41 | 409.19 | 0.64 | 0.60 |
| 7 | Chinese character reading (CCR) | 284 | 56.39 | 14.49 | 0.04 | -0.09 |
| 8 |  | 284 | 89.29 | 19.45 | 0.21 | -0.19 |
| 9 |  | 284 | 109.00 | 17.49 | -0.36 | -0.14 |
| 10 |  | 284 | 113.55 | 14.51 | -0.69 | 0.62 |
| 11 |  | 284 | 123.24 | 12.31 | -1.16 | 1.93 |
| 7 | Morphological production (MP) | 284 | 13.71 | 4.36 | 0.06 | -0.05 |
| 8 |  | 284 | 17.13 | 4.23 | -0.25 | -0.06 |
| 9 |  | 284 | 20.54 | 3.71 | -0.48 | 0.36 |
| 6 | Rapid number naming (RAN) | 284 | 16.95 | 5.39 | 0.97 | 1.14 |
| 7 |  | 284 | 12.20 | 3.54 | 1.00 | 1.37 |
| 8 |  | 282 | 10.17 | 2.60 | 1.07 | 1.53 |
| 9 |  | 284 | 8.54 | 2.23 | 1.11 | 1.88 |
| 6 | Orthography judgment (OJ) | 284 | 15.12 | 7.46 | 1.50 | 1.26 |
| 7 |  | 284 | 26.42 | 7.67 | -0.33 | -0.83 |
| 8 |  | 284 | 31.48 | 4.66 | -0.94 | 1.86 |
| 7* | Phoneme deletion (PD) | 284 | 19.11 | 0.17 | 2.91 | -5.34 |
| 8* |  | 284 | 19.61 | 0.09 | 1.55 | -8.50 |
| 9 |  | 284 | 18.71 | 0.11 | 1.79 | -1.70 |
| 7 | Tone detection (TD) | 284 | 17.18 | 4.95 | -0.37 | -0.82 |
| 8 |  | 284 | 17.98 | 4.99 | -0.59 | -0.62 |
| 9 |  | 284 | 19.69 | 4.42 | -1.15 | 0.51 |

* Kruskal Wallis Test was used for association test for skewed variables.
