# Supplementary material for: Association of DCDC2 Polymorphisms with Normal Variations in Reading Abilities in a Chinese Population
Source: PLoS One. 2016 Apr 21;11(4):e0153603. doi: 10.1371/journal.pone.0153603 (PMC4839751; doi:10.1371/journal.pone.0153603)
Supplement: S2 Table — (DOCX) [file pone.0153603.s004.docx]

Table S2 Pearson correlations between phenotype measures

|  |  | 1 | 2 | 3 | 4 | 5 | 6 | 7 | 8 | 9 | 10 | 11 | 12 | 13 | 14 | 15 | 16 | 17 | 18 | 19 | 20 | 21 | 22 | 23 | 24 | 25 |
| --- | --- | --- | --- | --- | --- | --- | --- | --- | --- | --- | --- | --- | --- | --- | --- | --- | --- | --- | --- | --- | --- | --- | --- | --- | --- | --- |
| 1 | Age 7 RF | -- |  |  |  |  |  |  |  |  |  |  |  |  |  |  |  |  |  |  |  |  |  |  |  |  |
| 2 | Age 8 RF | 0.84 | -- |  |  |  |  |  |  |  |  |  |  |  |  |  |  |  |  |  |  |  |  |  |  |  |
| 3 | Age 9 RF | 0.80 | 0.86 | -- |  |  |  |  |  |  |  |  |  |  |  |  |  |  |  |  |  |  |  |  |  |  |
| 4 | Age 10 RF | 0.75 | 0.81 | 0.85 | -- |  |  |  |  |  |  |  |  |  |  |  |  |  |  |  |  |  |  |  |  |  |
| 5 | Age 11 RF | 0.69 | 0.77 | 0.81 | 0.85 | -- |  |  |  |  |  |  |  |  |  |  |  |  |  |  |  |  |  |  |  |  |
| 6 | Age 7 CCR | 0.78 | 0.66 | 0.64 | 0.58 | 0.49 | -- |  |  |  |  |  |  |  |  |  |  |  |  |  |  |  |  |  |  |  |
| 7 | Age 8 CCR | 0.76 | 0.67 | 0.68 | 0.61 | 0.56 | 0.88 | -- |  |  |  |  |  |  |  |  |  |  |  |  |  |  |  |  |  |  |
| 8 | Age 9 CCR | 0.70 | 0.66 | 0.68 | 0.64 | 0.61 | 0.82 | 0.92 | -- |  |  |  |  |  |  |  |  |  |  |  |  |  |  |  |  |  |
| 9 | Age 10 CCR | 0.65 | 0.63 | 0.66 | 0.64 | 0.59 | 0.76 | 0.87 | 0.92 | -- |  |  |  |  |  |  |  |  |  |  |  |  |  |  |  |  |
| 10 | Age 11 CCR | 0.58 | 0.58 | 0.62 | 0.59 | 0.58 | 0.71 | 0.81 | 0.88 | 0.93 | -- |  |  |  |  |  |  |  |  |  |  |  |  |  |  |  |
| 11 | Age 7 MP | 0.56 | 0.50 | 0.50 | 0.49 | 0.45 | 0.55 | 0.59 | 0.56 | 0.55 | 0.49 | -- |  |  |  |  |  |  |  |  |  |  |  |  |  |  |
| 12 | Age 8 MP | 0.43 | 0.39 | 0.43 | 0.42 | 0.40 | 0.44 | 0.50 | 0.51 | 0.50 | 0.46 | 0.56 | -- |  |  |  |  |  |  |  |  |  |  |  |  |  |
| 13 | Age 9 MP | 0.46 | 0.40 | 0.45 | 0.46 | 0.46 | 0.44 | 0.50 | 0.57 | 0.55 | 0.54 | 0.48 | 0.58 | -- |  |  |  |  |  |  |  |  |  |  |  |  |
| 14 | Age 6 OJ | 0.36 | 0.23 | 0.20 | 0.14 | 0.12 | 0.43 | 0.39 | 0.29 | 0.24 | 0.18 | 0.15 | 0.15 | 0.06 | -- |  |  |  |  |  |  |  |  |  |  |  |
| 15 | Age 7 OJ | 0.39 | 0.35 | 0.33 | 0.30 | 0.23 | 0.52 | 0.53 | 0.49 | 0.50 | 0.45 | 0.32 | 0.31 | 0.30 | 0.36 | -- |  |  |  |  |  |  |  |  |  |  |
| 16 | Age 8 OJ | 0.35 | 0.33 | 0.34 | 0.32 | 0.28 | 0.44 | 0.47 | 0.48 | 0.47 | 0.46 | 0.29 | 0.28 | 0.33 | 0.15 | 0.53 | -- |  |  |  |  |  |  |  |  |  |
| 17 | Age 7 PD | 0.21 | 0.20 | 0.20 | 0.20 | 0.15 | 0.37 | 0.34 | 0.35 | 0.37 | 0.37 | 0.28 | 0.25 | 0.27 | -0.02 | 0.26 | 0.19 | -- |  |  |  |  |  |  |  |  |
| 18 | Age 8 PD | 0.14 | 0.16 | 0.13 | 0.18 | 0.18 | 0.28 | 0.22 | 0.25 | 0.29 | 0.33 | 0.18 | 0.24 | 0.31 | -0.02 | 0.21 | 0.29 | 0.43 | -- |  |  |  |  |  |  |  |
| 19 | Age 9 PD | 0.21 | 0.19 | 0.20 | 0.15 | 0.15 | 0.27 | 0.26 | 0.27 | 0.31 | 0.31 | 0.23 | 0.20 | 0.29 | -0.07 | 0.12 | 0.21 | 0.31 | 0.30 | -- |  |  |  |  |  |  |
| 20 | Age 7 TD | 0.26 | 0.24 | 0.23 | 0.24 | 0.27 | 0.33 | 0.37 | 0.35 | 0.41 | 0.43 | 0.29 | 0.23 | 0.34 | 0.09 | 0.26 | 0.21 | 0.32 | 0.24 | 0.29 | -- |  |  |  |  |  |
| 21 | Age 8 TD | 0.25 | 0.22 | 0.22 | 0.25 | 0.27 | 0.27 | 0.31 | 0.29 | 0.34 | 0.33 | 0.24 | 0.30 | 0.31 | 0.08 | 0.20 | 0.18 | 0.21 | 0.24 | 0.28 | 0.62 | -- |  |  |  |  |
| 22 | Age 9 TD | 0.26 | 0.26 | 0.26 | 0.29 | 0.29 | 0.35 | 0.37 | 0.39 | 0.41 | 0.43 | 0.25 | 0.31 | 0.36 | 0.04 | 0.23 | 0.25 | 0.24 | 0.17 | 0.39 | 0.55 | 0.61 | -- |  |  |  |
| 23 | Age 6 RAN | -0.49 | -0.45 | -0.43 | -0.41 | -0.35 | -0.52 | -0.48 | -0.44 | -0.45 | -0.41 | -0.36 | -0.35 | -0.31 | -0.25 | -0.32 | -0.23 | -0.21 | -0.16 | -0.24 | -0.24 | -0.25 | -0.30 | -- |  |  |
| 24 | Age 7 RAN | -0.47 | -0.45 | -0.46 | -0.43 | -0.40 | -0.44 | -0.45 | -0.46 | -0.45 | -0.44 | -0.38 | -0.40 | -0.34 | -0.14 | -0.29 | -0.26 | -0.25 | -0.24 | -0.26 | -0.35 | -0.31 | -0.29 | 0.61 | -- |  |
| 25 | Age 8 RAN | -0.43 | -0.45 | -0.43 | -0.43 | -0.42 | -0.37 | -0.41 | -0.43 | -0.41 | -0.43 | -0.38 | -0.35 | -0.36 | -0.08 | -0.21 | -0.18 | -0.15 | -0.19 | -0.20 | -0.27 | -0.30 | -0.30 | 0.61 | 0.74 | -- |
| 26 | Age 9 RAN | -0.37 | -0.35 | -0.38 | -0.36 | -0.38 | -0.33 | -0.37 | -0.43 | -0.44 | -0.42 | -0.31 | -0.30 | -0.32 | -0.10 | -0.20 | -0.21 | -0.15 | -0.20 | -0.18 | -0.20 | -0.20 | -0.22 | 0.55 | 0.672 | 0.76 |

*Note. RF-Reading fluency, CCR-Chinese character reading, MP-Morphological production, OJ-Orthography judgment, PD-Phoneme deletion, TD-Tone detection, RAN-Rapid number naming.*

*Correlation coefficients above 0.19 are significant at P < 0.001, correlation coefficients above 0.15 are significant at p < 0.01, and correlation coefficients above 0.12 are significant at p < 0.05, N = 284*
