# Supplementary material for: Association of DCDC2 Polymorphisms with Normal Variations in Reading Abilities in a Chinese Population
Source: PLoS One. 2016 Apr 21;11(4):e0153603. doi: 10.1371/journal.pone.0153603 (PMC4839751; doi:10.1371/journal.pone.0153603)
Supplement: S3 Table — (DOCX) [file pone.0153603.s005.docx]

Table S3. Estimated mean intercept, slope and model fitting estimates.

|  | Mean Intercept (*SE*) | Mean Slope (*SE*) | -2LL | *df* |
| --- | --- | --- | --- | --- |
| RF | 296.16 (10.00) | 217.64 (4.65) | 18720.04 | 10 |
| CCR | 79.16 (1.52) | 11.19 (0.28) | 10979.67 | 10 |

*Note*. RF-Reading fluency, CCR-Chinese character reading
