# Supplementary material for: Association of DCDC2 Polymorphisms with Normal Variations in Reading Abilities in a Chinese Population
Source: PLoS One. 2016 Apr 21;11(4):e0153603. doi: 10.1371/journal.pone.0153603 (PMC4839751; doi:10.1371/journal.pone.0153603)
Supplement: S4 Table — (DOCX) [file pone.0153603.s006.docx]

Table S4 Association of *rs807724* with reading and reading-related cognitive skills with outliers excluded

|  |  | N of outliers | Association test | | |
| --- | --- | --- | --- | --- | --- |
| Age | Phenotypic measurements |  | β^a,b^ | SE | *P* |
| 7 | Reading fluency (RF) | 14 | -0.395 | 0.135 | 0.004* |
| 8 |  | 11 | -0.474 | 0.161 | 0.003* |
| 9 |  | 4 | -0.377 | 0.175 | 0.032* |
| 10 |  | 5 | -0.531 | 0.17 | 0.002* |
| 11 |  | 7 | -0.407 | 0.165 | 0.014* |
| 7 | Chinese character reading (CCR) | 2 | -0.393 | 0.175 | 0.025* |
| 8 |  | 1 | -0.396 | 0.18 | 0.028* |
| 9 |  | 1 | -0.281 | 0.179 | 0.117 |
| 10 |  | 3 | -0.506 | 0.173 | 0.004* |
| 11 |  | 4 | -0.444 | 0.166 | 0.008* |
| 7 | Morphological production (MP) | 3 | -0.608 | 0.17 | 0.0004* |
| 8 |  | 5 | -0.694 | 0.172 | 0.0001* |
| 9 |  | 3 | -0.35 | 0.179 | 0.052 |
| 6 | Rapid number naming (RAN) | 8 | 0.533 | 0.167 | 0.002* |
| 7 |  | 9 | 0.466 | 0.16 | 0.004* |
| 8 |  | 11 | 0.46 | 0.156 | 0.003* |
| 9 |  | 11 | 0.176 | 0.158 | 0.266 |
| 6 | Orthography judgment (OJ)^c^ | 28 | 0.011 | 0.12 | 0.927 |
| 8 |  | 9 | -0.214 | 0.162 | 0.186 |
| 9 | Phoneme deletion (PD) | 11 | 0.036 | 0.153 | 0.815 |
| 9 | Tone detection (TD)^c^ | 9 | -0.562 | 0.163 | 0.001* |

*Note*. a, β represents standardized regression coefficient.

b, the minor allele was specified as the reference allele.

c, for OJ at age 7, TD at age 7 & 8, no outliers was found.

**, P*< 0.05.
